# Reduced Levels of NR1 and NR2A with Depression-Like Behavior in Different Brain Regions in Prenatally Stressed Juvenile Offspring 

**DOI:** 10.1371/journal.pone.0081775

**Published:** 2013-11-20

**Authors:** Hongli Sun, Lixia Guan, Zhongliang Zhu, Hui Li

**Affiliations:** 1 Department of Pharmacology, College of Medicine, Xi'an Jiaotong University, Shaanxi, China; 2 Department of Pharmacy, Liuzhou Municipal Liutie Central Hospital, Guangxi, China; 3 Shaanxi Province Biomedicine Key Laboratory, College of Life Sciences, Northwest University, Shaanxi, China; 4 Department of Neonatology, First Affiliated Hospital of Xi’an Jiaotong University, Shaanxi, China; Xi'an Jiaotong Univesity School of Medicine, China

## Abstract

Adolescence is a time of continued brain maturation, particularly in limbic and cortical regions, which undoubtedly plays a role in the physiological and emotional changes. Juvenile rats repeatedly exposed to prenatal stress (PS) exhibit behavioral features often observed in neuropsychiatric disorders including depression. However, to date the underlying neurological mechanisms are still unclear. In the current study, juvenile offspring rats whose mothers were exposed to PS were evaluated for depression-related behaviors in open field and sucrose preference test. NMDA receptor subunits NR1 and NR2A in the hippocampus, frontal cortex and striatum were assayed by western blotting. The results indicated that PS resulted in several behavioral anomalies in the OFT and sucrose preference test. Moreover, reduced levels of NMDA receptor subunits NR1 and NR2A in the hippocampus, and NR1 in prefrontal cortex and striatum of prenatally stressed juvenile offspring were found. Treatment with MK-801 to pregnant dams could prevent all those changes in the juvenile offspring. Collectivity, these data support the argument that PS to pregnant dams could induce depression-like behavior, which may be involved with abnormal expression of NR1 and NR2A in specific brain regions, and MK-801 may have antidepressant-like effects on the juvenile offspring.

## Introduction

Depression is a common and serious disorder of adolescence [1]. Lifetime prevalence increases dramatically from 1% of the population under age 12 to ~ 17%–25% of the population by the end of adolescence [2]. Prenatal stress (PS) in rats, like that in humans, has been shown to induce some psychiatric disorders including depressive-like behavior [3]. Alterations in glutamate neurotransmission are believed to play a role in the pathophysiology of depression [4]. As reports suggested that animals were exposed to either controllable or uncontrollable stressful events altered glutamate concentration in the hippocampus, prefrontal cortex [5], and striatum [6]. Moreover, brain changes associated with early-onset depression have been reported in the hippocampus, amygdala, caudate nucleus, putamen, and frontal cortex, structures that are extensively interconnected. They comprise a neuroanatomic circuit that has been termed the limbic-cortical-striatal-pallidal-thalamic tract. As the important components of this circuit, hippocampus, frontal cortex and striatum play an important role in the pathophysiology of depression [7,8]. 

PS can induce behavioral disturbances in animals [9]. The initial activity of a rat placed in novel surroundings has been taken as an indicator of its emotional state and the lack of acute activation in the OFT [10] and ahedonia in sucrose preference test [11] may bear some resemblance to depression. Relevant studies have reported the effects of antidepressant agents on open field behavior in rats [12,13], which demonstrated the validity of OFT used for measuring depression-like behavior. As to the sucrose preference test, which is often used to examine an animal’s depression-like behavior [14]. Our group has proven that PS significantly increased the glutamate concentration in hippocampal of juvenile offspring by high performance liquid chromatography [15]. Glutamate released from presynaptic neurons can interact with postsynaptic glutamate receptors, including N-methyl-D-aspartate receptors (NMDARs), kainate, and α–amino-3-hydroxyl-5-methyl-4-isoxazole propionate (AMPA). From several types of glutamate receptors, the contribution of the NMDARs to depression and antidepressant treatment is evidently demonstrated [16]. Three families of NMDA receptor subunits have been identified by molecular cloning: NR1, NR2 (A–D) and less commonly NR3 (A–B) subunits [17]. The receptor subunits follow a distinct developmental time-course. In rodents, the NR1 is present prenatally [18,19], low at birth, but progressively inclines the next 2-3 weeks [20]. The NR2A subunit, slowly increases throughout life [20]. NR1/NR2A receptors are mainly present at synaptic location and mediate fast neurotransmission [21]. However, it is not known whether PS alters the developmental profile of NMDA receptors. Early study showed the reduced expressions of the NR1 subunit in the spontaneously depressed Flinders Sensitive Line (FSL) rats, while maternal separation induced marked increases in the synaptic NR1 subunit in FSL rats [22]. NR2A-knockout mice showed antidepressant-like profiles in the forced swim test and tail suspension test in comparison with wild-type controls [23]. Meanwhile, NR2A-knockin mice with a Tyr-1325-Phe mutation showed antidepressant-like behaviors in the tail suspension test and forced swim test [24]. Taken together, these data appear to suggest abnormalities of NR1 and NR2A subunits of NMDA receptors in the pathophysiology of depression. 

A non-competitive antagonist of the NMDA receptor, (+)-5-methyl-10,11- dihydro-10-imine hydrogen maleate (MK-801), which crosses the blood–brain barrier [25] and undergoes placental transfer [26,27]. Studies have reported that MK-801 can produce antidepressant [28], and anticonvulsant effects [29]. McDonald et al. suggested the neuroprotective effects of MK-801 against NMDA induced neurotoxicity in vivo perinatal rat model [30]. However, there is controversy as to whether the drug has negative effects on the nervous system [31]. This may reflect differences in the doses and treatment regimen of MK-801 among the various studies. The MK-801 treatment dose used in the present study (0.2 mg/kg) is in the range of doses (0.05–1.0 mg/kg) that has been found to have a positive effect in the forced swim test in mice and rats [28,32,33]. In order to confirm the antidepressant-like effect of MK-801, we estimated the effect of MK-801 to verify that it could reverse PS-induced depression-like behavior in the juvenile offspring.

In this study, our aim was to test the depression-like behavior in the juvenile offspring with OFT and sucrose preference test. By using western blotting, we evaluated NR1 and NR2A alterations in the hippocampus, prefrontal cortex, and striatum for behavioral change, and further investigated the effect of the noncompetitive NMDA receptor antagonist MK-801 on PS-induced depression-like behavior in the juvenile offspring rats.

## Results

### Body weight

**Figure 1:**
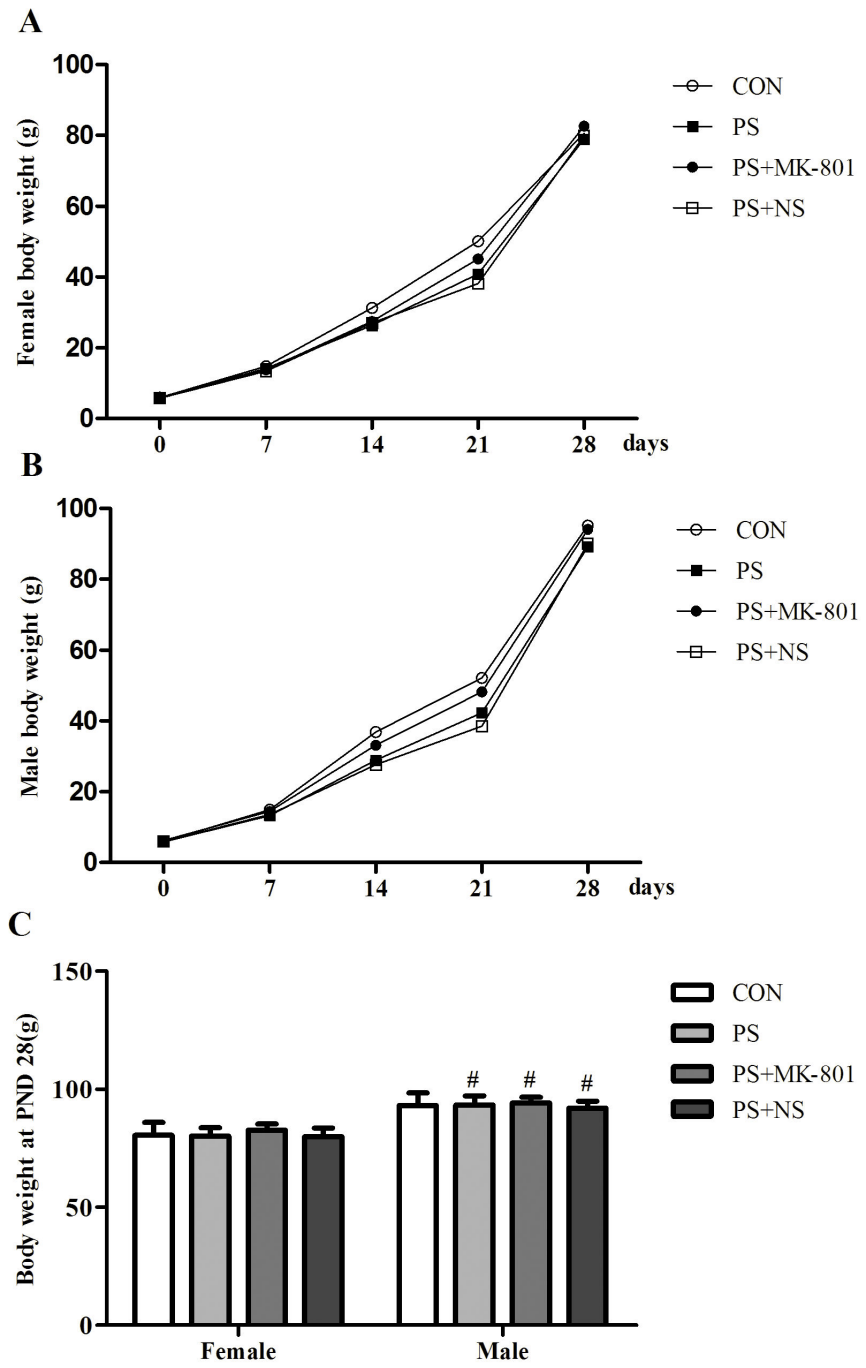
Effects of prenatal stress on the body weights of offspring. A: Weekly body weights of female offspring from PND 0 to PND 28. B: Weekly body weights of male offspring from PND 0 to PND 28. C: Body weights of both sexes on PND 28. Values represent means ± SEM. n=10 per group. **^*#*^** *p*<0.05 vs female.

Postnatal body weights of the offspring were shown in Figure 1 A, B and C. Both sexes offspring whose mother treated with PS or MK-801 did not present any significant changes of body weights on PND 0, 7, 14, 21, 28. Interestingly, sex difference in body weight was found on PND 28 (female < male, *p* < 0.05) in treated group, while CON group did not show this change.

### Depression-like behavior of juvenile offspring

#### Sucrose preference test

**Figure 2:**
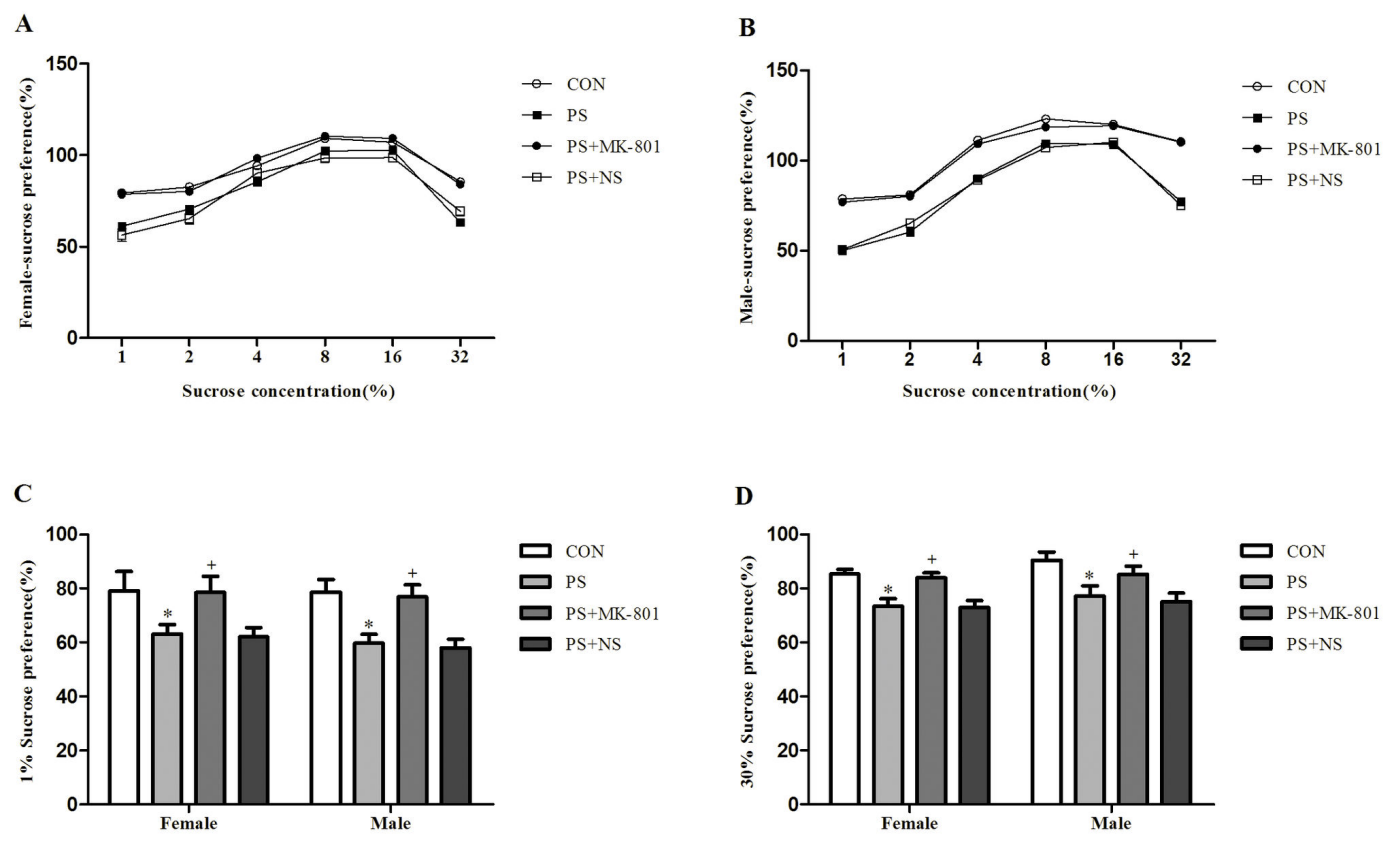
Effects of prenatal stress on the sucrose preference test. A: The percentage of sucrose consumed of female offspring at sucrose concentrations of 1 - 32%. B: The percentage of sucrose consumed of male offspring at sucrose concentrations of 1 - 32%. C: The percentage of sucrose consumed of both sexes at sucrose concentrations of 1%. D: The percentage of sucrose consumed of both sexes at sucrose concentrations of 32%. Values represent means ± SEM. n=10 per group. ^*^ *p*<0.05 vs CON, ^+^ *p*<0.05 vs PS+NS.

The values of the two-way ANOVA for the percentage of sucrose consumed are shown in Figure 2. The percentage of sucrose consumed was 1%≤2% < 4% < 8% = 16%>32 %, where < and >, but not ≤ indicated significant differences in all groups (*p* < 0.05) (Figure 2A: female, Figure 2B: male). There was a significant effect of PS treatment on the percentage of sucrose consumed with the sucrose concentration of 1% or 32% compared with CON group (*p* < 0.05) (Figure 2C: 1% , Figure 2D: 32%). No any significant effect of PS on the other sucrose concentration (2%, 4%, 8% or 16%) was found in the percentage of sucrose consumed (data not shown). In contrast, MK-801 treated subjects had a stronger sucrose preference than its matched controls at the sucrose concentration of 1% or 32% (*p* < 0.05). No sex difference in the percentage of sucrose consumed was found in the CON group or treatment group (PS or MK-801). No significant PS × gender or MK-801 × gender exposure interaction was found in the percentage of sucrose consumed. (Figure 2 A and B).

#### Open field test (OFT)

**Figure 3:**
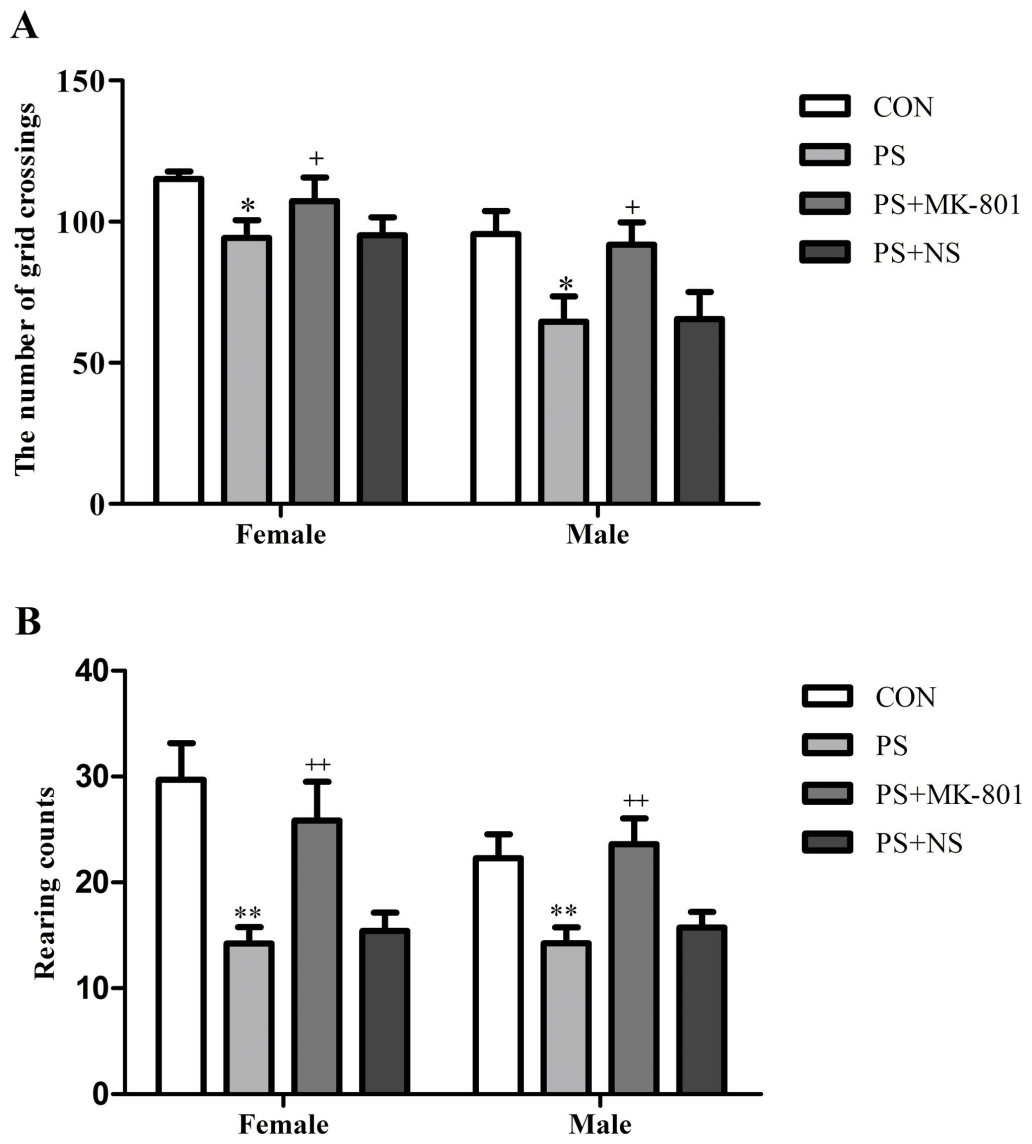
Effects of prenatal stress on the open field test. A: The number of grid crossings of both sexes. B: The rearing counts of both sexes. Values represent means ± SEM. n=10 per group. ^*^ *p*<0.05 vs CON, ^**^ *p*<0.01 vs CON, ^+^ *p*<0.05 vs CON, ^++^ *p*<0.01 vs PS+NS.

PS significantly decreased the number of grid crossings (*p* < 0.05), and the rearing counts (*p* < 0.01) in both sexes compared to their CON group respectively. However MK-801 significantly increased the number of grid crossings (*p* < 0.05), and the rearing counts (*p* < 0.01) in both sexes compared to their matched controls respectively. No sex difference in the number of grid crossings or the rearing counts was found in the CON group or treatment group (PS or MK-801). No significant PS × gender or MK-801 × gender exposure interaction was found in the number of grid crossings or the rearing counts by two-way ANOVA. (Figure 3 A and B ).

#### Expression of NR1 in the hippocampus, prefrontal cortex and striatum of juvenile offspring

**Figure 4:**
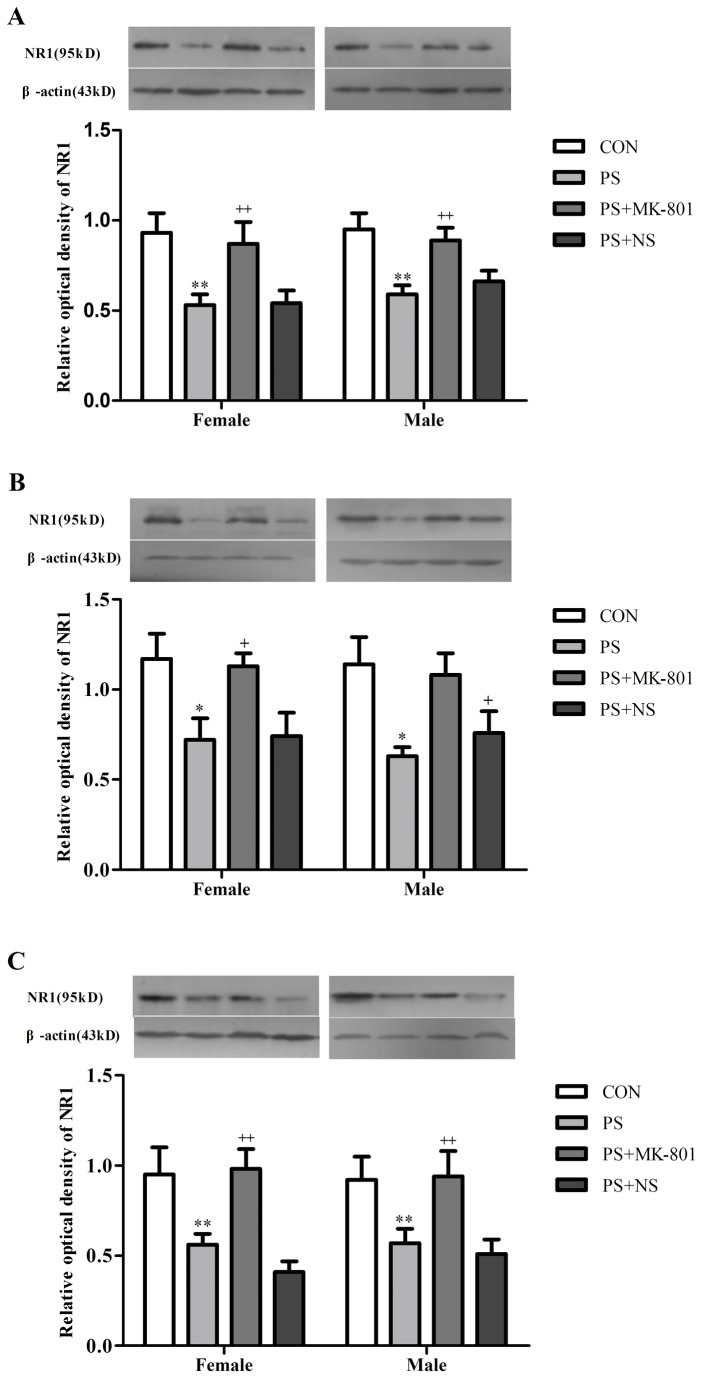
Effects of prenatal stress on the expression of NR1. A: NR1 levels in the hippocampus. B: NR1 levels in the frontal cortex. C: NR1 levels in the striatum. Values represent means ± SEM. n=10 per group. ^*^ *p*<0.05 vs CON, ^**^ *p*<0.01 vs CON, ^+^ *p*<0.05 vs CON, ^++^ *p*<0.01 vs PS+NS.

We found that the protein levels of NR1 showed a significant decrease following PS treatment in the hippocampus (*p* < 0.01), prefrontal cortex (*p* < 0.05) and striatum (*p* < 0.01) in both sexes. Whereas, this decrease of NR1 expression induced by PS was partially reversed by MK-801 treatment in the hippocampus (*p* < 0.01), prefrontal cortex (*p* < 0.05) and striatum (*p* < 0.01) in both sexes. No sex difference in the expression of NR1 in these three regions was found in the CON group or treatment group (PS or MK-801). No significant PS × gender or MK-801 × gender exposure interaction was found in the expression of NR1 in these three regions by two-way ANOVA. (Figure 4 A, B and C).

#### Expression of NR2A in the hippocampus, prefrontal cortex and striatum of juvenile offspring

**Figure 5:**
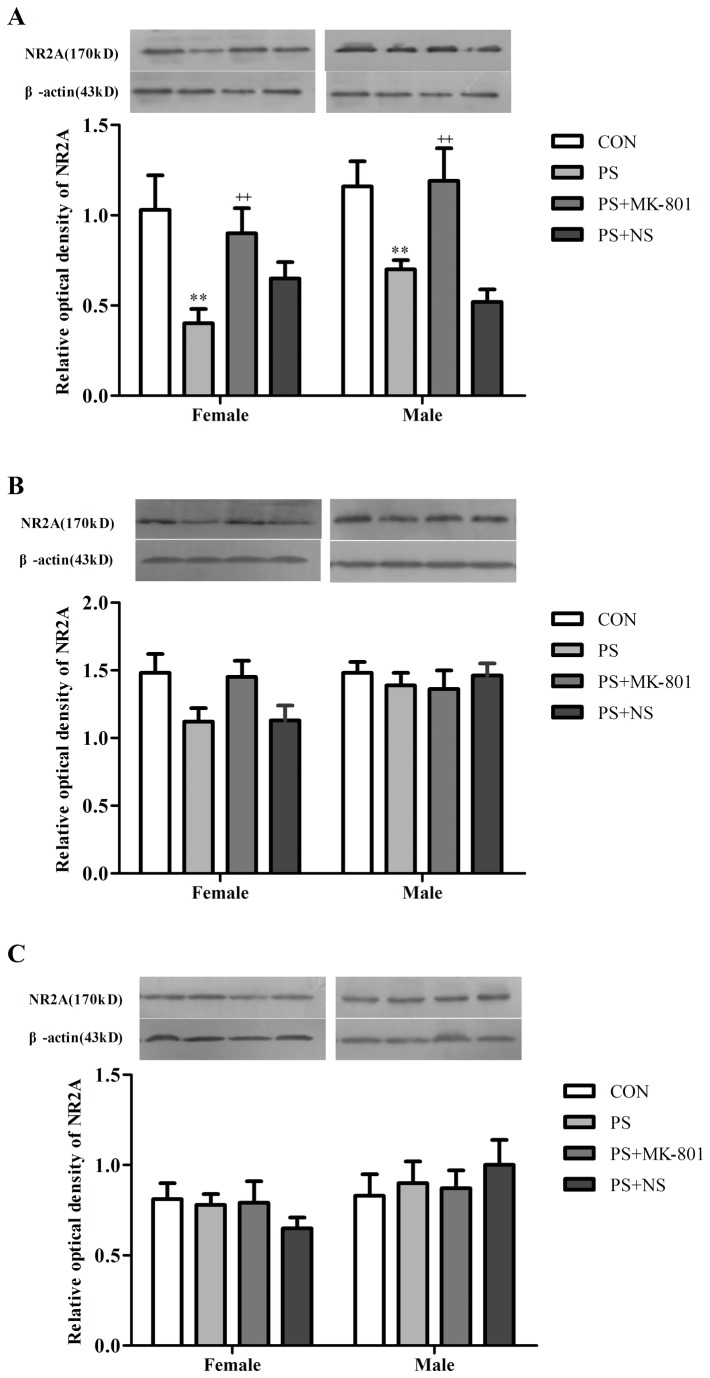
Effects of prenatal stress on the expression of NR2A. A: NR2A levels in the hippocampus. B: NR2A levels in the frontal cortex. C: NR2A levels in the striatum. Values represent means ± SEM. n=10 per group. ^**^p<0.01 vs CON, ^++^ *p*<0.01 vs PS+NS.

To investigate whether NR2A-containing NMDARs are involved in PS-induced depression, we examined the expression of NR2A in the hippocampus, prefrontal cortex and striatum by western blotting. As shown in (Figure 5 A, B and C), PS significantly lowered the expression of NR2A in the hippocampus (*p* < 0.01) in both sexes, while there was no changes of the expression of NR2A in the prefrontal cortex(*p* > 0.05) and striatum (*p* > 0.05) in both sexes. However, this decrease of NR2A expression induced by PS was partially reversed by MK-801 treatment in the hippocampus (*p* < 0.01) in both sexes. No sex difference in the expression of NR2A in these three regions was found in the CON group or treatment group (PS or MK-801). No significant PS × gender or MK-801 × gender exposure interaction was found in the expression of NR2A in these three regions by two-way ANOVA. 

## Discussion

In the present study, we demonstrated that chronic restraint stress given to pregnant dams could cause depression-like behavior in the juvenile offspring. Our present findings also suggested that NMDA receptors subunits NR1 and NR2A in a limited region in brain was reduced by PS. Furthermore, we found that MK-801 may have antidepressant-like effects on the juvenile offspring whose mother had been exposed to prenatal restraint stress during the last week of pregnancy. Sex difference in body weight (female < male) was found on PND 28 in the offspring of treated group, while CON group did not show this change, which however may be partly influenced by PS treatment to the mothers.

Many studies have indicated changes in behavior in depressed patients. In the present study we found that PS significantly decreased the number of grid crossings, and the rearing counts in the OFT. Surprisingly, the increased number of grid crossings and rearing counts induced by MK-801 in prenatally stressed offspring in comparison to normal saline treatment control was verified in the OFT. Depression-like behavior has been reported in findings using prenatally, chronic mild stressed rats in an OFT [34,35]. Furthermore, our previous study showed that enhanced depression-like behavior of prenatally stressed rats in the tail suspension test [8], and forced swimming test [36] in juvenile offspring rats, two tasks often used to examine an animal’s depression-like behavior [37]. Therefore, PS may be a good model of depression in animal, which is consistent with a study introduced animal models of depression [38]. We also found that the percentage of sucrose consumed, with the sucrose concentration of 1% or 32%, was reduced by PS, and solution intake increased and then decreased in all groups as a sucrose concentration increased from 1% to 32% in sucrose preference test. The volume of liquid intake was maximum at sucrose concentrations ranging from 8 and 16%. This confirms previous results obtained in Lister hooded rats [39], showing that sucrose drinking is related to the sucrose solution concentration according to an inverted U-shaped function, with maximal intake at the intermediate concentration (7%). Based on the study which reported that chronic mild stress induced an elevation of intracranial self-stimulation reward threshold in rats associated depression-like behavior [40], we speculated that PS may rise the sucrose preference threshold, which may partly explain why the percentage of sucrose consumed, with the sucrose concentration of 1% or 32%, was reduced by PS. Moreover, MK-801 prevented all those changes compared with saline treatment control. MK-801 blocks the neurophysiologic effects of the NMDA receptor complex by binding to a site in the cations channel of the receptor [29]. Different studies have shown that MK-801 possesses antidepressant properties. As Smolders et al reported that MK-801 enhanced motor activity in the OFT [41]. Besides, another study has also been shown that the stress-induced deficit in sucrose intake, which shows the depressive-behavior, was gradually reversed by chronic treatment with MK-801 [42]. The present results suggested that chronic restraint stress to pregnant dams could induce depression-like behavior, while MK-801 treatment to pregnant dams partially prevented the effect from depressive behavior on the juvenile offspring.

Abnormalities in the NMDA receptor system have been previously observed in postmortem brain tissue from major depressives and suicide victims [43]. NR1 is indispensable for all the constructions of NMDA receptors and distributes ubiquitously in the brain. The deficit with the expression of NR1 in the brain of olfactory bulbectomized (OB) rats, as a model of depression, indicated the decreased density and function of NMDA receptors [44]. Law and Deakin have reported that the expression of NR1 was decreased in the hippocampus of depressive patients [45]. Another study indicated a decrease in the amount of NR1 in the hippocampus and prefrontal cortex of OB rats [46]. In addition, a prior paper reported that there were no significant differences in the expression of NR1 in the striatum of depression patients compared with its matched controls [47]. On the other hand, some studies found that the competitive NMDA antagonist at glutamatergic sites (2-amino-7- phosphonoheptanoic acid; AP-7), a partial agonist at strychnine-insensitive glycine sites (1-aminocyclopropanecarboxyli acid) were active in the forced swim test and tail suspension test in mice [33,48]. A study also showed that MK-801, a non-competitive NMDA receptor antagonist, reduced immobility time in the forced swim test [28]. In the present study, the decreased amount of NR1 expression in the hippocampus, prefrontal cortex and striatum was found in prenatally stressed offspring. MK-801 treatment prevented this effect compared with its matched control. These findings further indicated a deficit of glutamatergic neurotransmission through NMDA receptors subunits NR1 in the depression-like behavior in juvenile offspring whose mothers were exposed to prenatal restraint stress during gestation.

Unlike NR1, the NR2 subunits region-restrictedly distribute in the brain. The NR2A distributes widely, but the levels of its expression are higher in the cerebral cortex, the hippocampus and cerebellar granule cells [46], and NR2 subunits require NR1 to form a functional complex [49]. In our present study, the significantly decreased NR2A expression was found in the hippocampus of prenatally stressed offspring rats. Another recently study suggested that PS induced significant increase in the amount of NR2A subunit at PND 28, but at PND 40-PND 60, the results showed a significant decrease of NR2A in the hippocampus as compare to the control pups [50]. These results showed the dynamic change of NR2A expression during the development of hippocampus in the offspring. In addition, no significant change of NR2A expression in prefrontal cortex of prenatally stressed offspring was found in the present study, which was different from those, Feyissa and Chandran revealed that there was a reduced expression of NR2A in the prefrontal cortex of depressed patients relative to controls [17]. In agreement with a study reported that there was no significant difference in the expression of NR2A in the striatum of depression patients [47], we also found no significant changes in the striatum of prenatally stressed offspring. This may be explained by a study which reported that stress impaired the hippocampus-dependent system and allowed the striatum to control behavior [51]. MK-801 partially prevented the decreasing of the NR2A expression in the hippocampus of prenatally stressed offspring in our present study. The result commendably reflected the antidepressant-like effects of NMDA receptor antagonists and partially enhanced the evidence of a study published about the rapid and robust antidepressant response in patients diagnosed with major depression to the ketamine (NMDA receptor antagonists) infusion [52]. Taken together, these findings highlighted that NMDA receptor signaling altered in depression may be related to the reduction of NR2A in the hippocampus. Furthermore, the antidepressant-like effects of MK-801showed in the present study may due to the reversion of such a reduction, however the specific mechanism of the action remains to be studied.

MK-801 binds selectively and with high affinity to NMDARs when they are in their open state[53]. There has been a large literatures on the effect of MK-801 on the level of NMDA receptor expression in rats. However, the underlying mechanism remains unknown. In previous studies examining NMDA-mediated excitotoxicity, there was a preferential increase of NR1 and NR2A mRNA after MK-801 administration in the cortex, striatum, and hippocampus, and it was determined through radio-ligand binding studies that pretreatment with MK-801 resulted in a higher number of NMDA binding sites[54]. In contrast, MK-801 has been reported to decrease NR1, NR2A and NR2B protein level in Pb2+-induced reductions of NMDA receptor subunits in vitro[55]. Moreover, the results of Zuo et al. indicated that the basal glutamate levels had a decreased tendency in the prefrontal cortex after MK-801 administration for 7 consecutive days [56]. MK-801 can also prevent the increase of glutamate utility[57]. These data may partly explain why MK-801 can increase the expression of NR1 or NR2. 

In Conclusion, the data reported herein implicated that PS induced depression-like behavior in the juvenile offspring with reduced the number of grid crossings and the rearing counts in the OFT. Besides, solution intake increased and then decreased in all groups as a sucrose concentration increased from 1% to 32% in sucrose preference test and less percentage of sucrose consumed with the sucrose concentration of 1% and 32% in the sucrose preference test was found in prenatally stressed offspring. Reduced levels of NMDA receptor subunits NR1 and NR2A in the hippocampus, and NR1 in prefrontal cortex and striatum of prenatally stressed juvenile offspring suggested an abnormality in NMDA receptors signaling in depression-like behavior. Moreover, the increased number of grid crossings and the rearing counts in prenatally stressed offspring in the OFT, and percentage of sucrose consumed with the sucrose concentration of 1% and 32% in the sucrose preference test induced by MK-801 treatment may be involved with a elevated expression of NR1 and NR2A in the hippocampus, and NR1 in prefrontal cortex and striatum. Such increasing of NR1 and NR2A expression in the specific brain regions may be involved of antidepressant-like effects of MK-801. Consequently, modification of NMDA receptor signaling represents a novel approach for the development of effective antidepressant medication.

## Materials and Methods

### Animals

**Figure 6:**
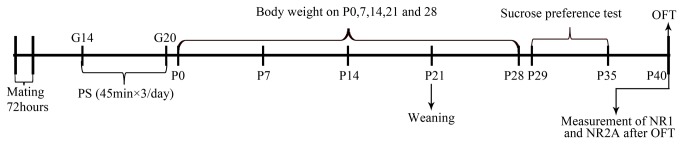
Timeline showing a summary of the experimental design. G: gestational age; P: postnatal age (days). PS: prenatal restraint stress; OFT: open field test.

The experimental time line is depicted in Figure 6. Sprague-Dawley rats were maintained at constant temperature (22°C) and humidity (60%) on a 12 h light/dark cycle (light on 08:00-20:00 h), freely accessing to food and water throughout the experiment. All procedures were carried out in accordance with the National Institutes of Health Guide for Care and Use of Laboratory Animals and were approved by the Institutional Animals Care and Use Committee at Xi’an Jiaotong University. Every effort was made to optimize comfort and to minimize the use of animals. Nulliparous female rats weighing 230-250g were housed with a sexually experienced male rat (280-350g) for mating (3:1), and the vaginal smear was examined on the following morning. The day on which the vaginal smear was positive was defined as day 0 of gestation. Each pregnant rat was then housed individually. 

### Procedure of prenatal stress

The PS model we used is restraint stress model [58]. The device was a transparent cylinder (6.8 cm in diameter) and the length could be adjusted to accommodate the size of the animals. Air holes of the cylinder were for breathing. The pregnant rats were randomly allocated to four groups: control (CON) group, PS group, PS+ normal saline (PS+NS) group, and PS+MK-801 group. The last three groups were subjected to restraint stress 3 times daily for 45 min on days 14-20 of pregnancy, and received administration of saline or MK-801(sigma, 0.2mg/kg, ip) 30min before stress. Mothers of the CON were undisturbed in their home cages during the same time period. To avoid animals from being habituated to the daily program, phases were randomly shifted within certain time periods (08:00 - 11:00, 11:00 - 14:00 and 16:00 - 19:00). After parturition, the pups were weighed on postnatal day (PND) 0, 7, 14, 21, 28 using a digital balance. All offspring rats were placed in the same plastic cage, and kept together with their natural mothers until weaning. The litters containing 8-14 pups with a suitable number of males and females were used for the experiment. After weaning, male and female pups were separated and housed four in each cage. On PND 30, one or two female and male pups were taken from each litter for the behavioral measures.

### Sucrose preference test

Ten male and ten female offspring rats from different dams were used for each group. Sucrose preference was assessed by a modified version of the sucrose preference test [59]. The sucrose consumption tests were performed using a two bottles test, rats having free access to both water and a sucrose solution. Animals were first trained to consume water in the two bottles and water consumption was measured for 24 h. The next day, sucrose consumption tests began: a bottle filled with a 1% sucrose solution replaced a bottle of water for 24 h. The next consumption tests were conducted with increasing concentrations of sucrose (i.e.: 2, 4, 8, 16 and finally 32% sucrose). Consumption was daily measured and each sucrose consumption test was separated by a water consumption test. Bottles were counterbalanced across the left and the right sides of the feeding compartment, and alternated in position from test to test. Sucrose preference (percent) was calculated as follows: preference = [sucrose solution intake (ml)/total fluid intake (ml)] × 100.

### Open field test (OFT)

Five days after the sucrose preference test. Both sexes were tested using a modified version of the OFT [10]. The open-field apparatus was a four-sided 80 cm × 80 cm × 40 cm wooden enclosure, with floor painted white and divided into 25 equal squares by black lines and side walls painted black. The central region of the box (3×3=9 squares) was subdivided into a large center and a small center of 8 and 1 squares respectively. Tests were conducted in a darkened, quiet room lit by two 40-W light bulbs suspended above the center of the open field. Each rat was gently placed onto the small center of the open-field apparatus facing away from an observer and allowed to explore freely for 5 min. During the test time, the animal's movements in the field were quantified by conuting the number of grid crossings (at least three paws in a square) and the rearing counts (wall rears and free rears). After each animal was tested, the test apparatus was cleaned with a solution of 70% alcohol to eliminate odors for the next test.

### Western blotting

The rats were anesthetized with 20% ethylcarbamate (1.2-1.5mL/100g, i.p.), and decapitated within 30 min after behavior tests ended. The hippocampus, prefrontal cortex and striatum tissues were dissected on the petridish filled with ice and immediately frozen in liquid nitrogen, and then kept in a﹣80℃ freezer until analysis. HIP, PFC and STR sample were homogenized in 1000μL ice-cold Tris-HCl buffer (10mM, pH 7.4), containing 1% SDS, 0.1mM EDTA, 0.1mM EGTA, 0.1mM PMSF (Sigma, USA) (B1), Samples were centrifuged at 1000g for 10 min at 4 °C to remove nuclei and large debris (P1). The supernatant (S1) was then transferred to another tube and centrifuged again at 18,000g for 30min at 4°C to obtain a clarified fraction of cytosolic and light membrane fraction (S2) and a pellet corresponding to the crude synaptosomal fraction (P2), which was then resuspended in the same buffer and centrifuged at 25,000g to obtain a synaptosomal membrane fraction (LP1). Finally, the pellet was resuspended in 100μL the same buffer (B1) added with 320mM sucrose. Protein concentrations were determined using a Nano Drop ND-1000 spectrophotometer (Nano Drop, USA). The protein was conserved in a sterile 1.5mL Eppendorf tube in -80°C refrigerator. Before loading the SDS-PAGE gel, 25μL 5× loading buffer was added into each sample protein and then denaturized in 60°C water-bath for 1h. Then equal amounts of protein from the HIP, PFC and STR were loaded onto 10% SDS-PAGE gel. After electrophoresis, the protein were transferred to 0.45μm polyvinyldifluoride (PVDF) membranes (Millipore, USA), which were then blocked with Blotto (5% free-fat dry milk in 0.01M PBS containing 0.1% Tween 20) for 2h at room temperature. The PVDF membrane was incubated with rabbit polyclonal to NMDAR1 antibody (1:1000, ab52177, Abcam, USA) or polyclonal to NMDAR2A antibody (1:1000, ab14596, Abcam, USA) in PBST buffer overnight at 4°C. The PVDF membrane was rinsed with PBST three times (10min/time) and incubated in goat anti-rabbit secondary antibody (1:8000, Pioneer, China) for 1h at room temperature with gentle shaking, immunoreactivity was detected with an ECL Western Blotting Detection Kit (Millipore, USA). Results were standardized to β-actin CON protein, which was detected by evaluating the band density at 43 kD after probing with a mouse anti-rat polyclonal antibody (1:10000, Pioneer, China). The lanes were calculated by the Quantity One software. 

### Statistics

All values reported were mean ± SEM. Offspring data analyses were explored by two-way ANOVA (PS × gender or MK-801 × gender) using the software SPSS 13.0. When appropriate, Post-Hoc Least Significant Difference (LSD) test was used to analyse the multiple comparisons. A difference was considered significantly at p<0.05 level. 
